# Supplementary material for: High-Throughput Effect-Directed Monitoring Platform for Specific Toxicity Quantification of Unknown Waters: Lead-Caused Cell Damage as a Model Using a DNA Hybrid Chain-Reaction-Induced AuNPs@aptamer Self-Assembly Assay
Source: Sensors (Basel). 2023 Aug 3;23(15):6877. doi: 10.3390/s23156877 (PMC10422636; doi:10.3390/s23156877)
Supplement: Supplementary file 1 [file sensors-23-06877-s001.zip › sensors-2475283-supplementary.pdf]

Supplementary Information

High throughput effect- directed monitoring platform for specific toxicity quantification of unknown waters: lead caused mitochondrial damage as a model using DNA Hybrid chain reaction induced AuNPs@aptamer self-assembly assay.

*Jiaxuan Xiao‡[1], Kuijing Yuan‡[2], Yu Tao[1], Yuhan Wang[1], Xiaofeng Yang[1], Jian Cui[3], Dali Wei[1], Zhen Zhang[1]**

[1] School of Emergency Management, School of the Environment and Safety Engineering, Jiangsu University, Zhenjiang 212013, China.

[2]Dalian Center for Food and Drug Control and Certification, Dalian 116037, China.

[3]Institute of Botany, Jiangsu Province and Chinese Academy Sciences (Nanjing Botanical Garden Mem. Sun Yat-Sen), Nanjing 210014, China;

*Corresponding author:

E-mail: zhangzhen@ujs.edu.cn

Fax: +86-511-88790955

SUPPLEMENTARY FIGURE CAPTIONS:

Figure S1. The AFM image of HCR products.

Figure S2. The depth histogram of HCR products.

Figure S3. TEM images of AuNPs.

Figure S4. TEM images of AuNPs nanoflare.

Figure S5. Effect of the ratio of initiator to hairpin (A), the concentration of Linker (B), the HCR reaction time (C), the combination time of AuNPs@aptamer@Linker DNA and HCR products (D), the incubation time between aptamer and ATP. Error bars = RSD (n=3).

SUPPLEMENTARY TABLE CAPTIONS:

Table S1. The DNA sequences used for this method**.**

Table S2. Detailed information of sampling sites in 2020.

Figure S1. AFM image of HCR products

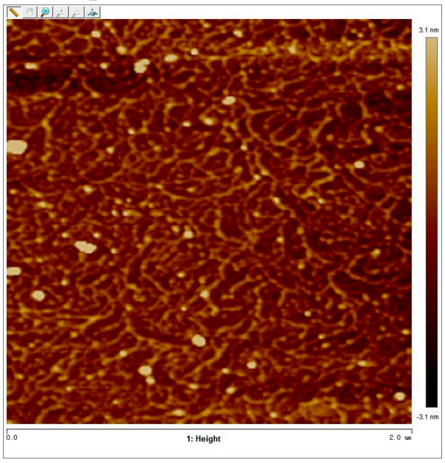

Figure S2. Depth histogram of HCR products.

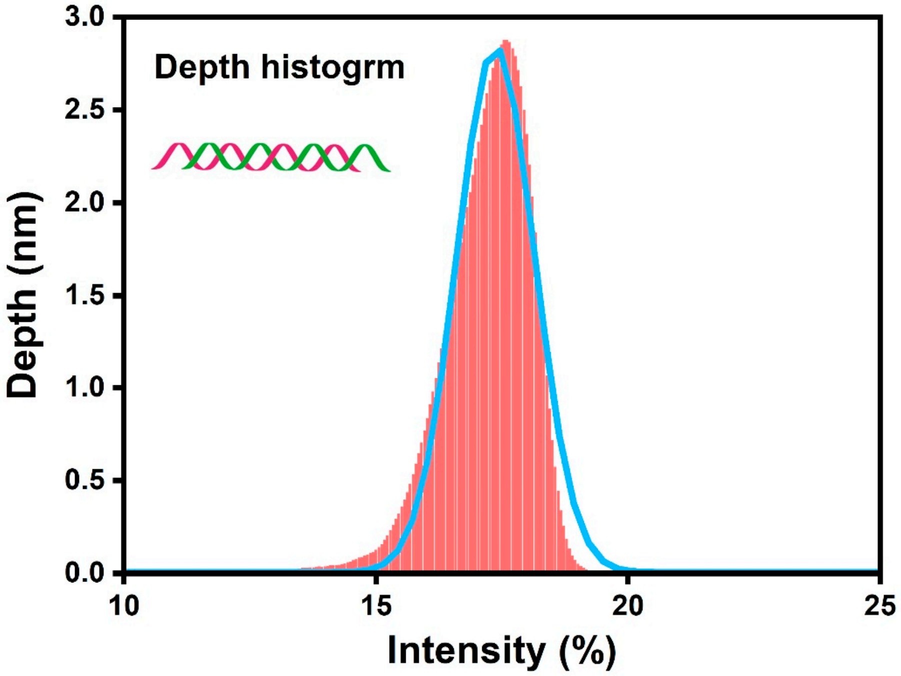

Figure S3. TEM images of AuNPs.

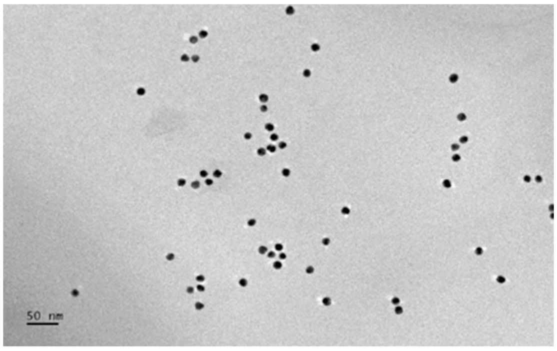

Figure S4. TEM images of single AuNPs nanoflare.

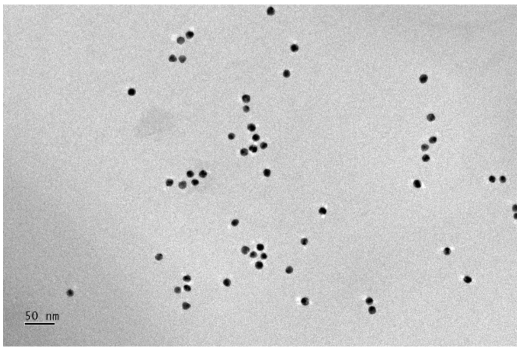

Figure S5. Effect of the ratio of initiator to hairpin (A), the concentration of Linker (B), the HCR reaction time (C), the combination time of AuNPs@aptamer@Linker DNA and HCR products (D), the incubation time between aptamer and ATP. Error bars = RSD (n=3).

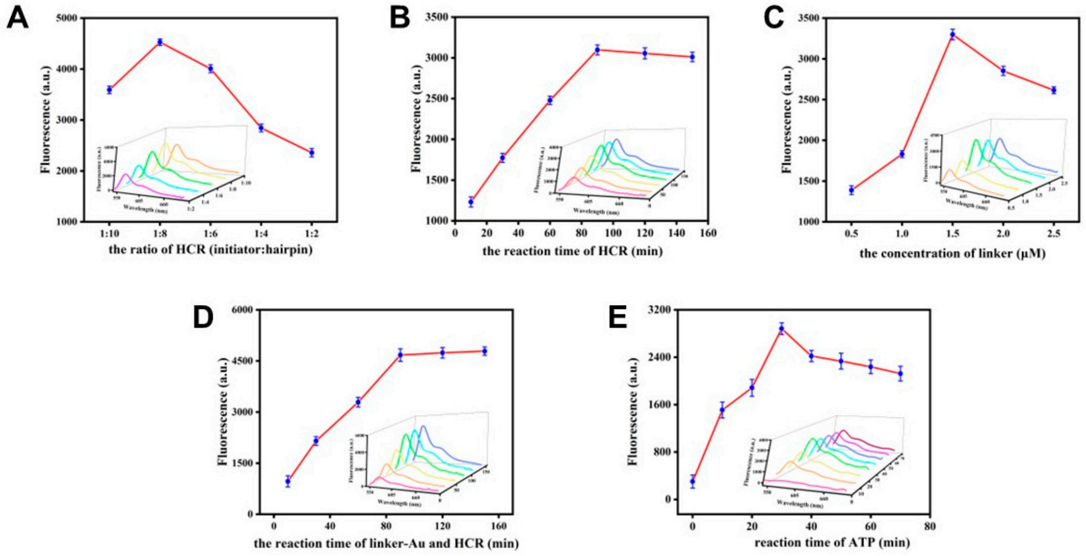

Table S1. Details of the DNA sequences.

ATP aptamer	5′- ACCTGGGGGAGTATTGCGGAGGAAGGTGTCACA -SH-$(CH_2)_6$-3′
Blocking strand	5′- Cy3 TGTGACACCTTCCT -3′
Linker DNA	5′- SH-(CH2)6-T(30)GTTCGTAGCGCCATTCTG -3′
Initiator	5′- TCTCAAGGACCACCGCATCTCTAC -3′
Hairpin 1	5′- GTAGAGATGCGGTGGTCCTTGAGACAAAGTTCTCAAGGACCACCGCATTTTCAGAATGGCGCTACGAAC -3′
Hairpin 2	5′- TCTCAAGGACCACCGCATCTCTACATGCGGTGGTCCTTGAGAACTTTG -3′

Table S2. Detailed information of sampling sites in 2020.

Sample No.	Site	Long	Lat
S1	Yunliang River Qilidian Bridge	E119°24'53"	N32°11'31"
S2	Yunliang River Barrage	E119°23'20"	N32°12'24"
S3	Yunliang River Runzhoushanlu Bridge	E119°24'43"	N32°12'24"
S4	Yunliang River Taiping Bridge	E119°25'1"	N32°12'42"
S5	Yangtze River Zhengrunzhou Wharf	E119°24'21"	N32°14'14"
S6	Jinshan Scenic Spot	E119°25'24"	N32°13'20"
S7	Grand Canal Inlet	E119°26'7"	N32°13'8"
S8	Grand Canal Jingkou Barrage	E119°27'3"	N32°13'5"
S9	Grand Canal Huju Bridge	E119°28'7"	N32°12'4"
S10	Grand Canal Zhoujia River	E119°28'47"	N32°11'32"
S11	Grand Canal Dingmao Bridge	E119°29'10"	N32°11'20"
S12	Grand Canal City No.1 Middle school	E119°29'34"	N32°11'19"
S13	Grand Canal Tuanjie River	E119°31'4"	N32°11'23"
S14	Grand Canal Miaojiawanlu Bridge	E119°30'26"	N32°11'6"
S15	Grand Canal Dantu Barrage	E119°32'7"	N32°11'52"
S16	Grand Canal Shanghuanglu Bridge	E119°32'29"	N32°10'31"
S17	Grand Canal Panjia Village	E119°33'11"	N32°9'59"
S18	Beijing-Hangzhou Canal upstream	E119°33'43"	N32°10'38"
S19	Beijing-Hangzhou Canal Tributary	E119°37'23"	N32°11'6"
S20	Beijing-Hangzhou Canal downstream	E119°33'23"	N32°11'6"
S21	Yangtze River Tributary Binshui Road	E119°27'1"	N32°13'12"
S22	Yangtze River Tributary Linjiang Bridge	E119°28'4"	N32°13'57"
S23	Yangtze River Tributary Jiaoshan Tail	E119°29'37"	N32°14'3"
S24	Yangtze River upstream	E119°21'45"	N32°12'5"
S25	Yangtze River downstream	E119°35'24"	N32°11'3"
